# Supplementary material for: Evolutionary origins and innovations sculpting the mammalian PRPS enzyme complex
Source: bioRxiv. 2024 Oct 1:2024.10.01.616059. Preprint. [Version 1] doi: 10.1101/2024.10.01.616059 (PMC11476008; doi:10.1101/2024.10.01.616059)
Supplement: Supplement 7 [file NIHPP2024.10.01.616059v1-supplement-7.pdf]

bioRxiv preprint doi: https://doi.org/10.1101/2024.10.01.616059; this version posted October 1, 2024. The copyright holder for this preprint (which was not certified by peer review) is the author/funder, who has granted bioRxiv a license to display the preprint in perpetuity. It is made available under aCC-BY 4.0 International license.

Supplementary Fig. 1

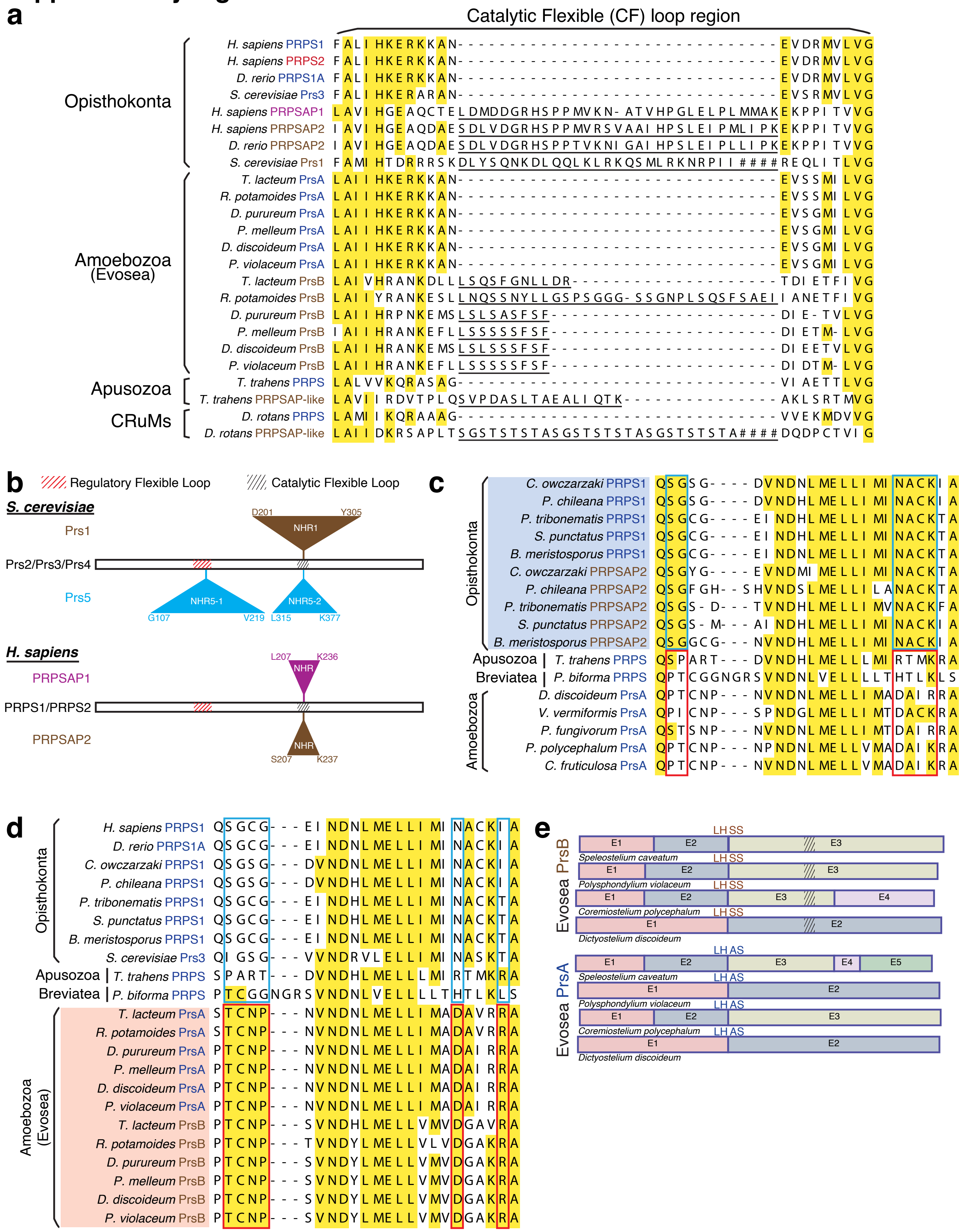

bioRxiv preprint doi: https://doi.org/10.1101/2024.10.01.616059; this version posted October 1, 2024. The copyright holder for this preprint (which was not certified by peer review) is the author/funder, who has granted bioRxiv a license to display the preprint in perpetuity. It is made available under aCC-BY 4.0 International license.

Supplementary Figures and Tables

Supplementary Fig.1: Convergent evolution of PRPSAP-like homologs throughout Amorphea via gene duplications from PRPS1-encoding genes

a) The catalytic flexible (CF) loop from the sequence alignments of PRPS homologs from representative organisms of Opisthokonta, Amoebozoa (Evosea), Apusozoa, and CRuMs. PrsA in Evosea is the PRPS enzyme while PrsB represents the PRPS homologs with insertions in the CF loop (NHRs), similar to Opisthokonta PRPSAP2. PRPS homologs in Apusozoa and CRuMs that share features with Opisthokonta PRPSAP2 are termed “PRPSAP-like”. Underlined residues denote the NHRs. For *S. cerevisiae* Prs1 and *D. rotans* PRPSAP-like sequence, only a part of the insertion is shown, and the rest of the sequence is represented by hatch marks.

b) PRPS paralogs in *Saccharomyces cerevisiae* and *Homo sapiens* showing relative positions of non-homologous regions – NHR1 of Prs1, NHR5-1 and NHR5-2 of Prs5, NHRs of PRPSAP1 and PRPSAP2. The open bar represents the polypeptide for Prs2, Prs3, and Prs4 in *S. cerevisiae*, and for PRPS1 and PRPS2 in *H. sapiens*. The insertion points for Prs1, Prs5, PRPSAP1, and PRPSAP2 are marked, with NHRs indicated as triangles either above or below the open bar. The residue numbers on the NHRs indicate the amino acid positions on the respective homologs.

c) and d) The N-terminal residues from a sequence alignment of PRPS homologs from representative organisms of Amorphea. Opisthokonta PRPSAP2 sequences are more similar to Opisthokonta PRPS enzymes (blue box) than to PRPS from Apusozoa, Breviatea and Amoebozoa (c) while Evosea PrsB sequences are more similar to Evosea PrsA (red box) than to PRPS from Apusozoa, Breviatea and Opisthokonta (d) indicating that PRPSAP2 and PrsB likely emerged independently from ancestral Opisthokonta PRPS1 and Evosea PrsA, respectively.

bioRxiv preprint doi: https://doi.org/10.1101/2024.10.01.616059; this version posted October 1, 2024. The copyright holder for this preprint (which was not certified by peer review) is the author/funder, who has granted bioRxiv a license to display the preprint in perpetuity. It is made available under aCC-BY 4.0 International license.

e) Analysis of conserved splice site junction among PrsA and PrsB homologs across different representative organisms of Evosea. Exons from each of the Prs encoding genes are merged to highlight the splice site junctions. PrsA shares a conserved splice site junction – LH_(A/S)S – with PrsB, further supporting that PrsB likely originated from a gene duplication event in the ancestral Evosea PrsA.

bioRxiv preprint doi: https://doi.org/10.1101/2024.10.01.616059; this version posted October 1, 2024. The copyright holder for this preprint (which was not certified by peer review) is the author/funder, who has granted bioRxiv a license to display the preprint in perpetuity. It is made available under aCC-BY 4.0 International license.

Supplementary Fig. 2

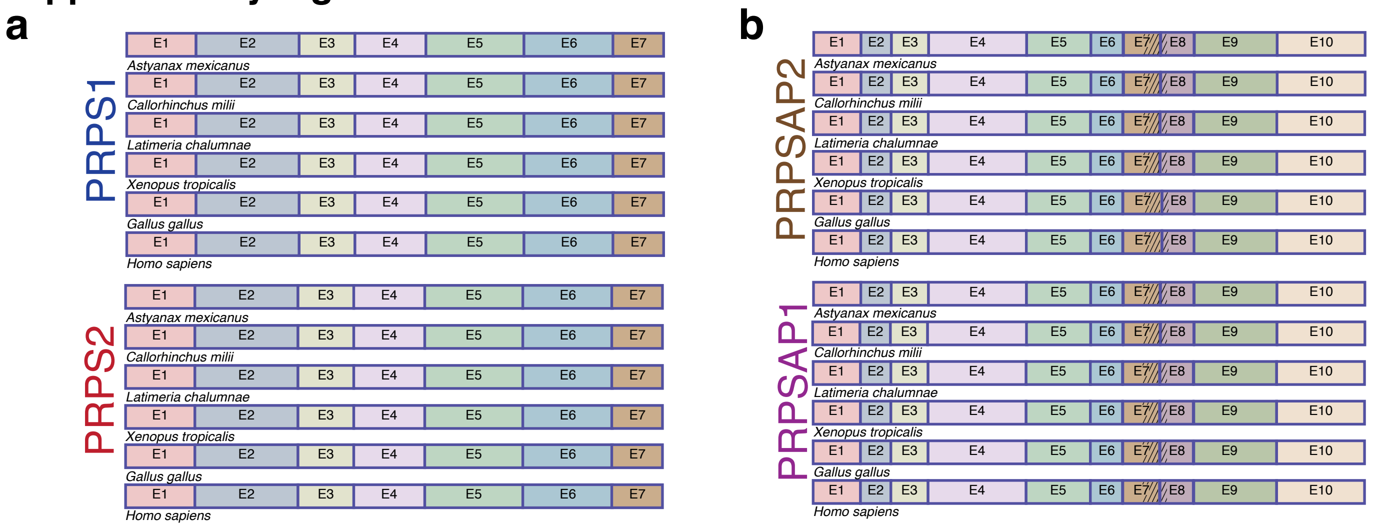

bioRxiv preprint doi: https://doi.org/10.1101/2024.10.01.616059; this version posted October 1, 2024. The copyright holder for this preprint (which was not certified by peer review) is the author/funder, who has granted bioRxiv a license to display the preprint in perpetuity. It is made available under aCC-BY 4.0 International license.

Supplementary Fig.2: PRPS2 and PRPSAP1 emerged in the ancestor of jawed vertebrates from PRPS1 and PRPSAP2, respectively.

a) Analysis of splice site junctions among PRPS1 and PRPS2 across different representative organisms of jawed vertebrates. Exons from each of the PRPS1 and PRPS2 encoding genes are merged to highlight the highly conserved splice site junctions.

b) Analysis of splice site junction among PRPSAP2 and PRPSAP1 across different representative organisms of jawed vertebrates. Exons from each of the PRPSAP2 and PRPSAP1 encoding genes are merged to highlight the highly conserved splice site junctions. Hatch marks representing the NHRs are not intended to accurately represent the variable length of the NHRs.

bioRxiv preprint doi: https://doi.org/10.1101/2024.10.01.616059; this version posted October 1, 2024. The copyright holder for this preprint (which was not certified by peer review) is the author/funder, who has granted bioRxiv a license to display the preprint in perpetuity. It is made available under aCC-BY 4.0 International license.

Supplementary Fig.3

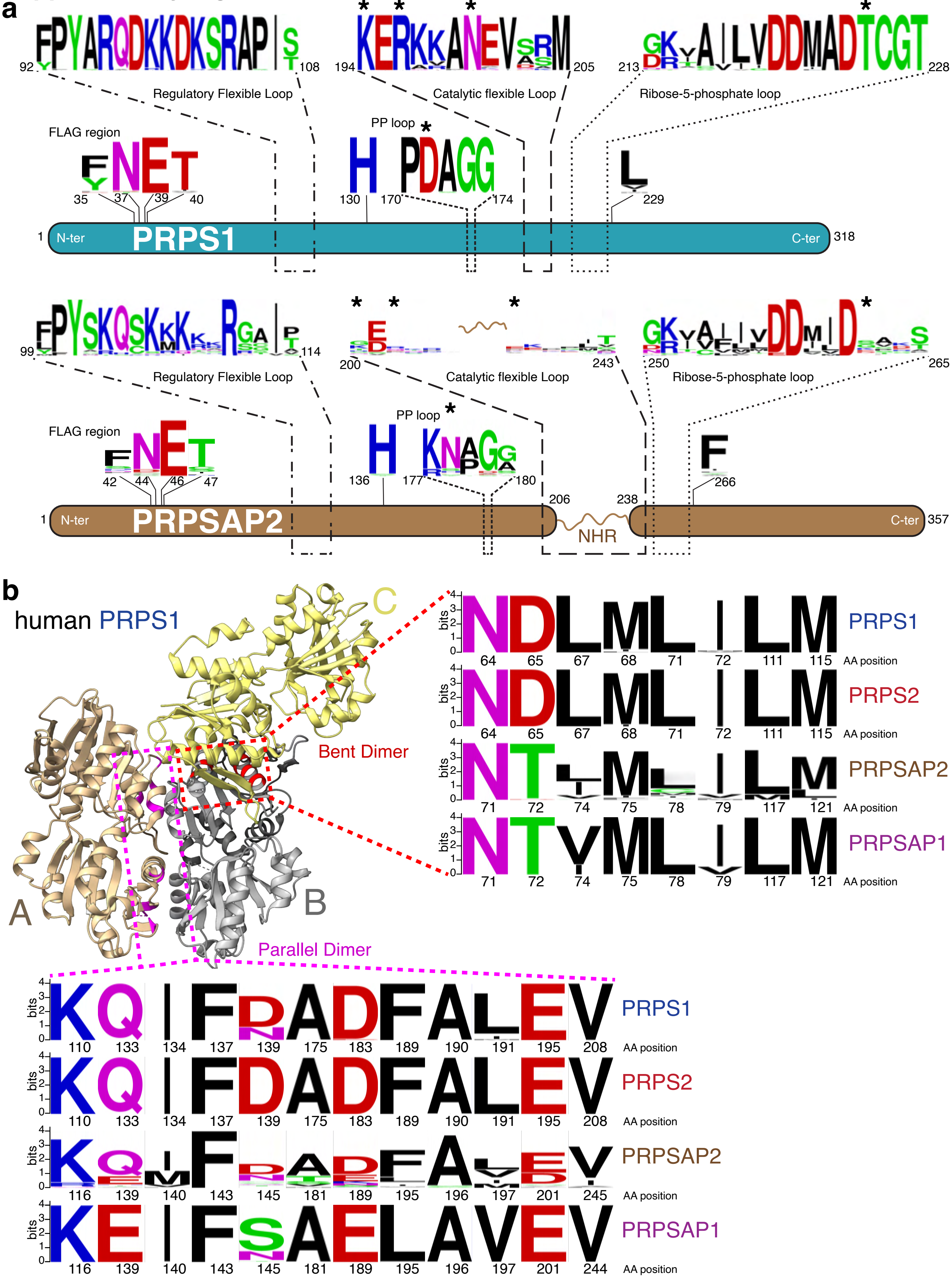

bioRxiv preprint doi: https://doi.org/10.1101/2024.10.01.616059; this version posted October 1, 2024. The copyright holder for this preprint (which was not certified by peer review) is the author/funder, who has granted bioRxiv a license to display the preprint in perpetuity. It is made available under aCC-BY 4.0 International license.

Supplementary Fig.3: Opisthokont PRPSAP homologs have non-conserved loop regions critical for catalysis but conserved dimer interfaces.

a) WebLogo depicting the multiple sequence alignment of the active site residues of PRPS1 and PRPSAP2 from representative organisms in Opisthokonta (n = 44 for PRPS1 and n = 44 for PRPSAP2). Residues from the FLAG region, regulatory flexible loop, pyrophosphate (PP) loop, CF loop, and R5P binding loop are indicated. Many residues shown are conserved in PRPS1 but not in PRPSAP2. The numbers below the logo sequences indicate the corresponding residue positions of the human PRPS1 (NP_002755.1) and PRPSAP2 (NP_001340030.1). Asterisks denote some evolutionarily conserved active site residues that are exclusive to PRPS enzymes and highlighted in Fig.1(d) but replaced with different residues at the corresponding positions in PRPSAPs, highlighted in Fig.1(e).

b) The trimeric structure of human PRPS1 (PDB: 2HCR). In the dashed box, red- and magenta-colored residues represent those involved in the formation of bent (B and C subunits) and parallel (A and B subunits) dimers, respectively. The amino acid sequence of *B. subtilis* PRPS[15] was aligned with the Opisthokonta PRPS homologs, and the corresponding dimer interface residues were selected for generating the WebLogo. Sequences for PRPS1 (n = 44) and PRPSAP2 (n = 44) are derived from representative organisms within opisthokonts, while PRPS2 (n = 46) and PRPSAP1 (n = 92) sequences are derived from representative organisms within jawed vertebrates. The numbers below the logo sequences indicate the corresponding residues positions of the human PRPS1 (NP_002755.1), PRPS2 (NP_002756.1), PRPSAP1 (AAH09012.1), and PRPSAP2 (NP_001340030.1). The significant sequence conservation between PRPS and PRPSAP indicates the potential for heteromeric associations.

bioRxiv preprint doi: https://doi.org/10.1101/2024.10.01.616059; this version posted October 1, 2024. The copyright holder for this preprint (which was not certified by peer review) is the author/funder, who has granted bioRxiv a license to display the preprint in perpetuity. It is made available under aCC-BY 4.0 International license.

Supplementary Fig. 4

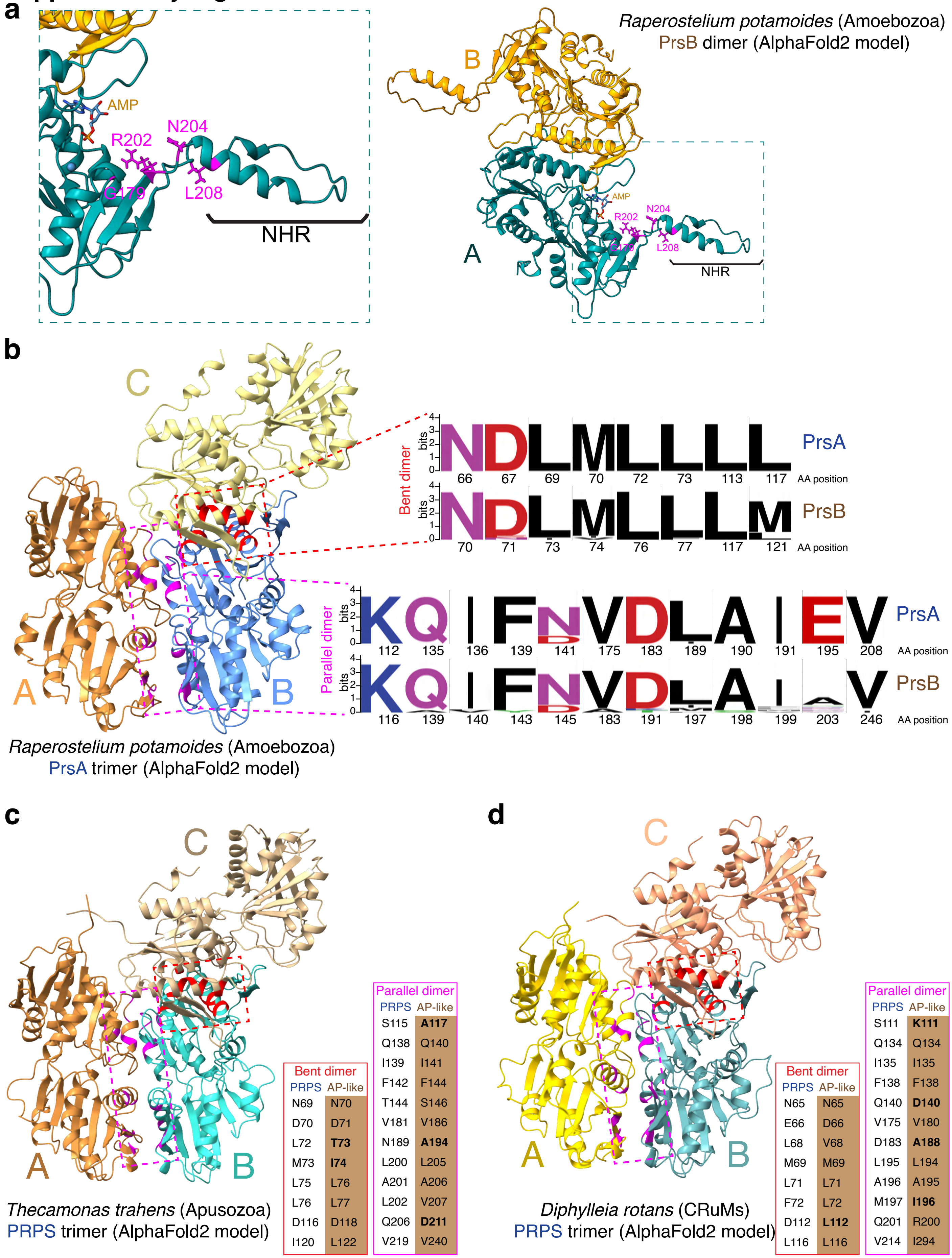

Panel c – Bent dimer

PRPS	AP-like
N69	N70
D70	D71
L72	**T73**
M73	**I74**
L75	L76
L76	L77
D116	D118
I120	L122

Panel c – Parallel dimer

PRPS	AP-like
S115	**A117**
Q138	Q140
I139	I141
F142	F144
T144	S146
V181	V186
N189	**A194**
L200	L205
A201	A206
L202	V207
Q206	**D211**
V219	V240

Panel d – Bent dimer

PRPS	AP-like
N65	N65
E66	D66
L68	V68
M69	M69
L71	L71
F72	L72
D112	**L112**
L116	L116

Panel d – Parallel dimer

PRPS	AP-like
S111	**K111**
Q134	Q134
I135	I135
F138	F138
Q140	**D140**
V175	V180
D183	**A188**
L195	L194
A196	A195
M197	**I196**
Q201	R200
V214	I294

bioRxiv preprint doi: https://doi.org/10.1101/2024.10.01.616059; this version posted October 1, 2024. The copyright holder for this preprint (which was not certified by peer review) is the author/funder, who has granted bioRxiv a license to display the preprint in perpetuity. It is made available under aCC-BY 4.0 International license.

Supplementary Fig.4: PRPSAP-like homologs in non-opisthokont Amorphea lineages are likely non-catalytic with conserved dimer interfaces.

a) Predicted dimeric structure of *R. potamoides* PrsB (annotated from SRX8374346-9) from the AlphaFold2 model, and a zoom in highlights four non-conserved residues in PrsB (magenta) at the corresponding positions of residues for PrsA (Table 1). AMP was modeled into the dimer to denote the putative ATP binding site. NHR represents the insertion in the CF loop.

b) Predicted trimeric structure of *R. potamoides* PrsA (annotated from SRX8374346-9) from the AlphaFold2 model. In the dashed box, red- and magenta-colored residues represent those involved in the formation of bent (B and C subunits) and parallel (A and B subunits) dimers, respectively. The amino acid sequence of *B. subtilis* PRPS[15] was aligned with the Amoebozoa PRPS homologs, and the corresponding dimer interface residues were selected for generating the WebLogo. Sequences for PrsA (n = 19) and PrsB (n = 20) are derived from representative organisms within Amoebozoa. The numbers below the logo sequences indicate the corresponding residues positions of *R. potamoides* PrsA and PrsB. Significant sequence conservation between PrsA and PrsB indicates the potential for heteromeric associations.

c) and d) represents the predicted trimeric structure of *T. trahens* (representative organism of Apusozoa) PRPS (XP_013753676.1) in (c) and *D. rotans* (representative organism of CRuMs) PRPS (annotated from SRX3153023) in (d) from the AlphaFold2 model. In the dashed box, red- and magenta-colored residues represent those involved in the formation of bent (B and C subunits) and parallel (A and B subunits) dimers, respectively. A comparison of dimer interface residues at similar positions in PRPSAP-like is shown. Non-conserved residues in PRPSAP-like relative to PRPS are shown in bold. Significant sequence conservation between PRPS and PRPSAP-like indicates the potential for heteromeric associations.

bioRxiv preprint doi: https://doi.org/10.1101/2024.10.01.616059; this version posted October 1, 2024. The copyright holder for this preprint (which was not certified by peer review) is the author/funder, who has granted bioRxiv a license to display the preprint in perpetuity. It is made available under aCC-BY 4.0 International license.

Supplementary Fig. 5

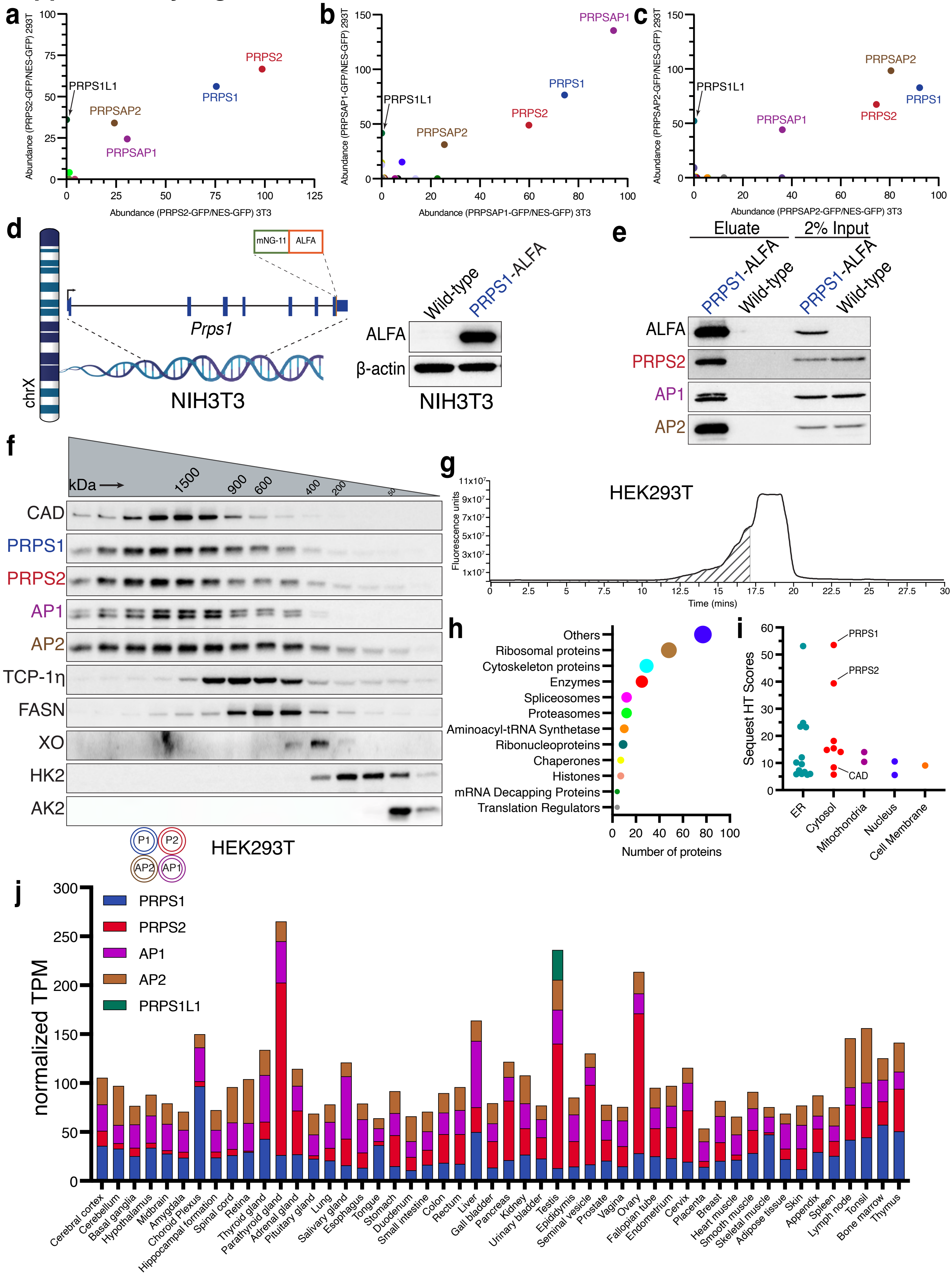

bioRxiv preprint doi: https://doi.org/10.1101/2024.10.01.616059; this version posted October 1, 2024. The copyright holder for this preprint (which was not certified by peer review) is the author/funder, who has granted bioRxiv a license to display the preprint in perpetuity. It is made available under aCC-BY 4.0 International license.

Supplementary Fig.5: The stable PRPS complex is one of the largest cytosolic metabolic assemblies in cells.

a) and b) and c) represent the scatter plots from mass spectrometry (MS) runs of eluates from GFP IP in stably expressing PRPS2-GFP, PRPSAP1-GFP, and PRPSAP2-GFP, respectively in NIH3T3 (x-axis) and HEK293T (y-axis) cells. The axes represent the square root-transformed SEQUEST HT scores normalized to control.

d) Schematic of *Prps1* gene with an endogenous ALFA tag knocked in frame at the C-terminus. mNG-11 represents the short fragment of the split monomeric Neon Green protein and ALFA is an epitope tag containing residues – PSRLEEELRRRLTEP. Western blot validating full-length expression of the endogenously tagged PRPS1-ALFA protein is shown.

e) ALFA pulldown from the whole cell extracts of knock-in NIH3T3 cells demonstrating the interaction of endogenous PRPS1 with PRPS2, PRPSAP1, and PRPSAP2.

f) Western blot analysis of SEC fractions collected from HEK293T native whole cell lysates. Cell lysates were fractionated on a Superose 6 Increase 3.2/300 column. Immunoblots probing PRPS complex members and internal standards are shown. In the pictogram, a double circle means multiple copies of the protein are interacting within the heteromeric complex.

g) Chromatogram showing SEC traces of HEK293T whole cell lysates fractionated on a Bio SEC-5 2000Å column, which offers better fractionation for proteins/protein complexes in high molecular weight (HMW) range. The proteins that eluted in the fractions shown as hatch marks were sent for mass-spectrometry analyses for identification of proteins in HMW range.

h) Classification of HMW proteins based on their functions (manual curation) from the mass-spectrometry dataset obtained from fractions collected in (g).

i) Subclassification of enzymes from (h) based on their cellular localization.

bioRxiv preprint doi: https://doi.org/10.1101/2024.10.01.616059; this version posted October 1, 2024. The copyright holder for this preprint (which was not certified by peer review) is the author/funder, who has granted bioRxiv a license to display the preprint in perpetuity. It is made available under aCC-BY 4.0 International license.

j) Normalized transcript per million (nTPM) of PRPS complex components in various human tissues obtained from Human Protein Atlas.

bioRxiv preprint doi: https://doi.org/10.1101/2024.10.01.616059; this version posted October 1, 2024. The copyright holder for this preprint (which was not certified by peer review) is the author/funder, who has granted bioRxiv a license to display the preprint in perpetuity. It is made available under aCC-BY 4.0 International license.

Supplementary Fig. 6

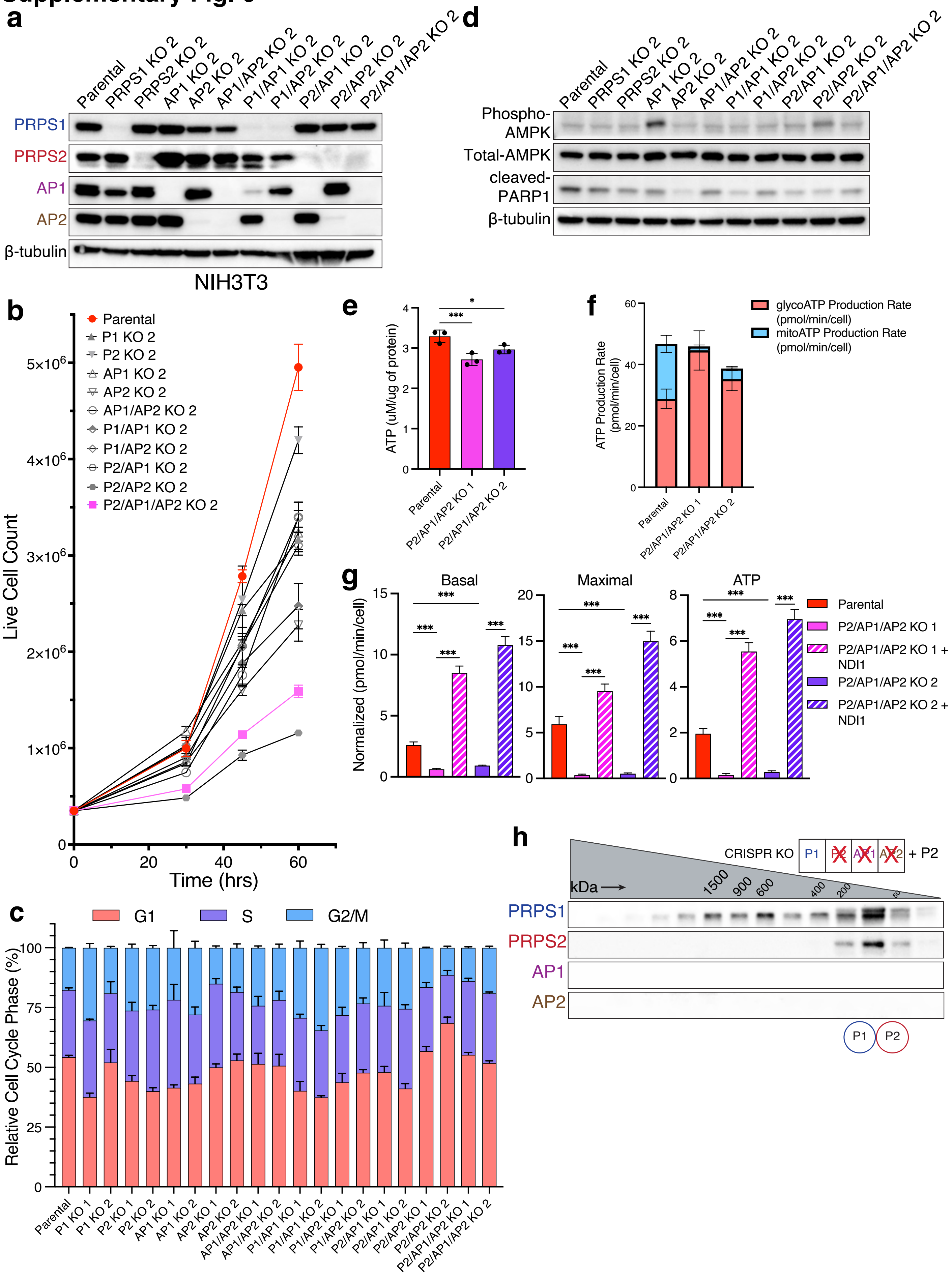

bioRxiv preprint doi: https://doi.org/10.1101/2024.10.01.616059; this version posted October 1, 2024. The copyright holder for this preprint (which was not certified by peer review) is the author/funder, who has granted bioRxiv a license to display the preprint in perpetuity. It is made available under aCC-BY 4.0 International license.

Supplementary Fig.6: Additional phenotypic characterization of NIH3T3 isogenic knockout lines

a) Western blot validation of the second set of CRISPR-Cas9-generated isogenic knockout cell lines.

b) Proliferation for the panel of NIH3T3 knockout cell lines generated in a). Error bars represent SD; n = 3.

c) Bar graph depicting propidium-iodide-based cell cycle profiles: G1, S, and G2/M phases for the panel of NIH3T3 knockout cell lines. Error bars represent SD; n = 3.

d) Immunoblots were probed with phospho-AMPK (T172) and cleaved PARP1 antibody in the panel of NIH3T3 knockout cell lines as readouts for energy stress and apoptosis, respectively.

e) Total cellular ATP (normalized to the protein content) measured by ATP determination assay in NIH3T3 parental and P2/AP1/AP2 KO cell lines. Error bars represent SD; n = 3. (*P < 0.05; ***P < 0.001 by one-way ANOVA post hoc test).

f) ATP production rate determined by Seahorse ATP Rate assay in NIH3T3 parental and P2/AP1/AP2 KO cell lines. Error bars represent SD; n = 7.

g) Quantification of basal respiration, maximal respiration (post FCCP injection), and respiration coupled to ATP production measured by Seahorse ATP Rate Assay in NDI1 expressing P2/AP1/AP2 KO cell lines. Error bars represent SD; n = 7. (***P < 0.001 by one-way ANOVA post hoc test).

h) Western blot analysis of SEC fractions collected from NIH3T3 P2/AP1/AP2 KO native whole cell lysates transiently transfected with PRPS2. Cell lysates were fractionated on a Superose 6 Increase 3.2/300 column. In the pictogram, a single circle means a single protein is interacting within the complex.

bioRxiv preprint doi: https://doi.org/10.1101/2024.10.01.616059; this version posted October 1, 2024. The copyright holder for this preprint (which was not certified by peer review) is the author/funder, who has granted bioRxiv a license to display the preprint in perpetuity. It is made available under aCC-BY 4.0 International license.

Supplementary Fig. 7

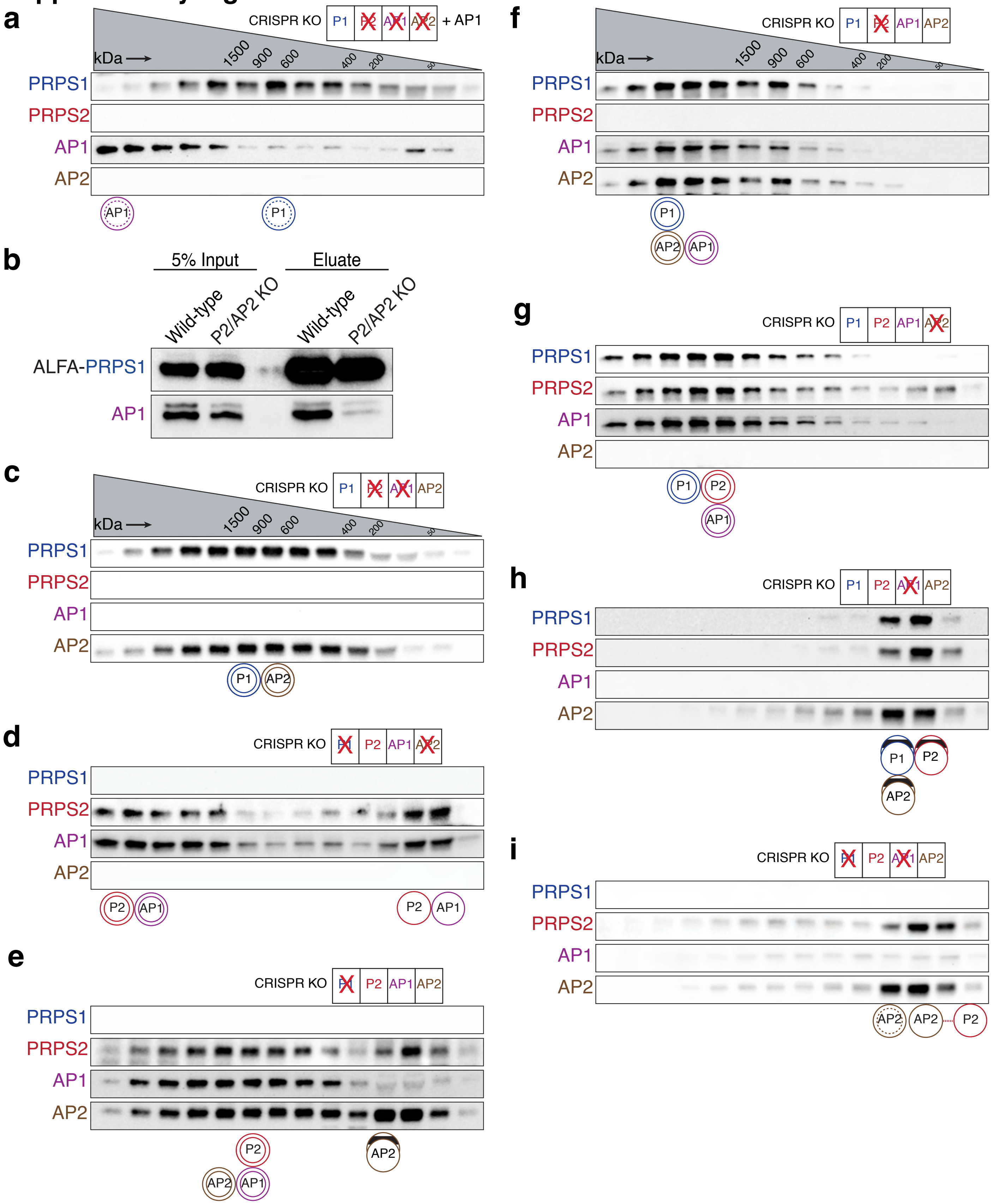

bioRxiv preprint doi: https://doi.org/10.1101/2024.10.01.616059; this version posted October 1, 2024. The copyright holder for this preprint (which was not certified by peer review) is the author/funder, who has granted bioRxiv a license to display the preprint in perpetuity. It is made available under aCC-BY 4.0 International license.

Supplementary Fig.7: SEC profiles of additional isogenic knockout lines

a) Western blot analysis of SEC fractions collected from native whole cell lysates of NIH3T3 P2/AP1/AP2 KO cells stably expressing AP1

b) ALFA pulldown from the whole cell extracts of NIH3T3 parental and P2/AP2 KO cells transiently transfected with PRPS1-ALFA

c-i) Western blot analysis of SEC fractions collected from native whole cell lysates of NIH3T3 P2/AP1 KO cells (c), P1/AP2 KO cells (d), P1 KO cells (e), P2 KO cells (f), AP2 KO cells (g), AP1 KO cells (h), and P1/AP1 KO cells (i).

All the fractionation experiments were performed on Superose 6 Increase 3.2/300 column. The circular pictograms at the bottom of the SEC immunoblots illustrate the different configurations of the PRPS complex. A double circle means multiple copies of the protein are interacting within the heteromeric complex. A double circle with a dotted inner circle means multiple copies of the protein are forming homo-oligomers. A single circle means a single protein is interacting within the complex. A circle with lines inside indicates that the proteins may be forming a trimer or tetramer.

bioRxiv preprint doi: https://doi.org/10.1101/2024.10.01.616059; this version posted October 1, 2024. The copyright holder for this preprint (which was not certified by peer review) is the author/funder, who has granted bioRxiv a license to display the preprint in perpetuity. It is made available under aCC-BY 4.0 International license.

Supplementary Fig. 8

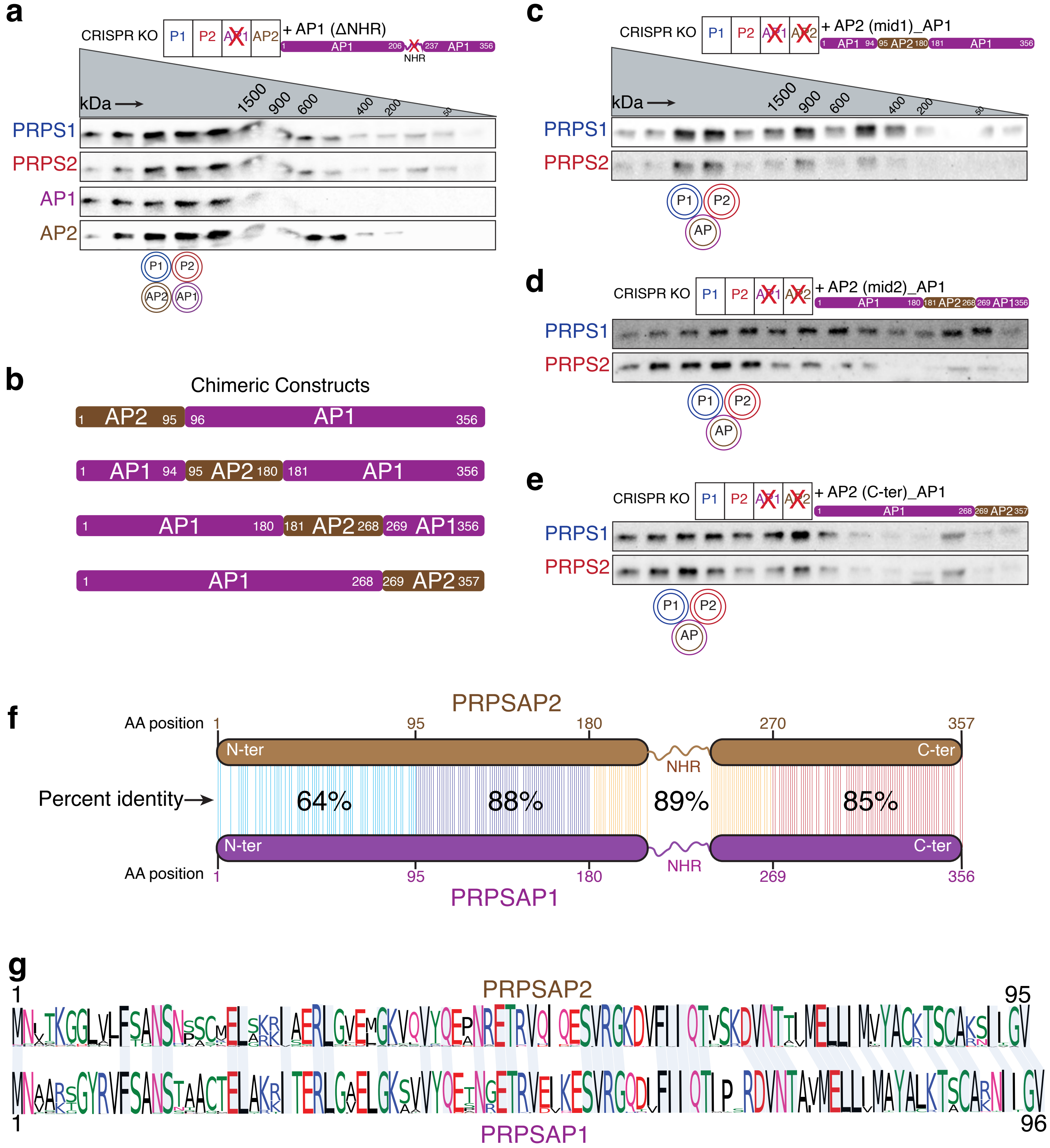

bioRxiv preprint doi: https://doi.org/10.1101/2024.10.01.616059; this version posted October 1, 2024. The copyright holder for this preprint (which was not certified by peer review) is the author/funder, who has granted bioRxiv a license to display the preprint in perpetuity. It is made available under aCC-BY 4.0 International license.

Supplementary Fig.8: SEC profiles of chimeric PRPSAP1/AP2-expressing cells

a) Western blot analysis of SEC fractions collected from native whole-cell lysates of NIH3T3 AP1 KO cells stably expressing AP1 lacking the non-homologous region (NHR). The absence of NHR in AP1 does not prevent AP1-mediated complex elongation.

b) Schema of chimeric constructs created to test AP1-specific domains that confer complex elongation property.

c-e) Western blot analysis of SEC fractions collected from native whole-cell lysates of NIH3T3 AP1/AP2 KO cells stably expressing chimeric AP1 constructs: one containing residues 95-180 from AP2 (b), residues 181-268 from AP2 (c), and AP2's C-terminus (residues 269-357) (d).

f) Comparison of human AP1 and AP2 amino acid sequence. The four regions switched in Fig.4 (e) and Supplementary Figs. 8 (c)-(e) are highlighted with different colors. Each line connecting AP1 and AP2 represents identical amino acids at that position. The regions spanning residues 1-95 show the greatest variation, with only 64% amino acid identity. The poorly conserved, highly variable amino acids present in the NHRs were excluded from this analysis.

g) MetaLogo depicting the multiple sequence alignment of the N-terminal amino acid residues of AP1 and AP2 from representative organisms of jawed vertebrates (n = 92 for AP1 and n = 93 for AP2). Residue numbers for AP1 and AP2 correspond to the human homologs (AAH09012.1 and NP_001340030.1, respectively).

All the fractionation experiments were performed on Superose 6 Increase 3.2/300 column. In the pictogram, a double circle means multiple copies of the protein are interacting within the heteromeric complex.

bioRxiv preprint doi: https://doi.org/10.1101/2024.10.01.616059; this version posted October 1, 2024. The copyright holder for this preprint (which was not certified by peer review) is the author/funder, who has granted bioRxiv a license to display the preprint in perpetuity. It is made available under aCC-BY 4.0 International license.

Supplementary Fig. 9

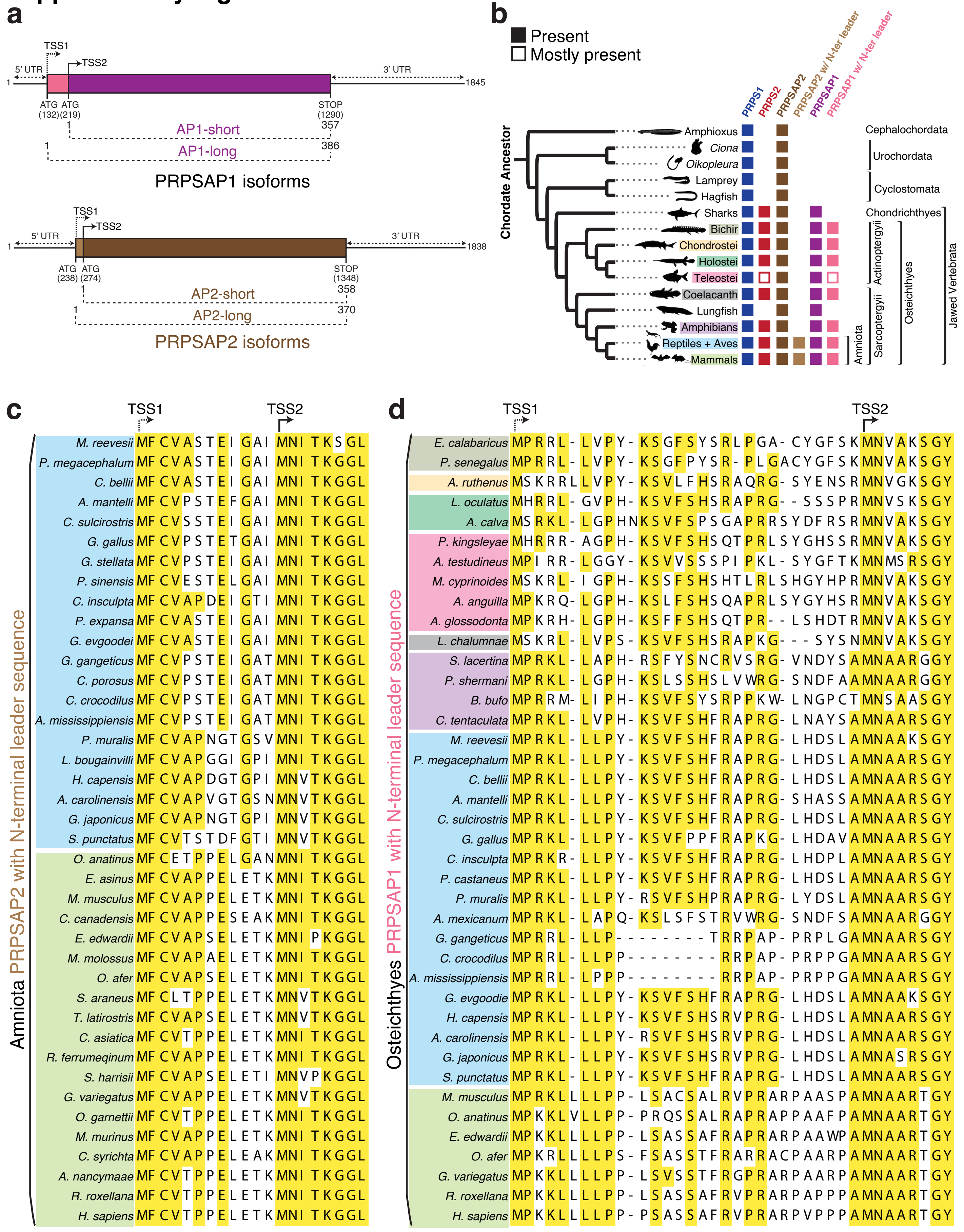

bioRxiv preprint doi: https://doi.org/10.1101/2024.10.01.616059; this version posted October 1, 2024. The copyright holder for this preprint (which was not certified by peer review) is the author/funder, who has granted bioRxiv a license to display the preprint in perpetuity. It is made available under aCC-BY 4.0 International license.

Supplementary Fig.9: Conservation of upstream translation start site in PRPSAPs

a) Schematic representation of alternative start sites in mammalian AP1 and AP2 and their consequent translation into the short and long isoforms. Base positions for AP1 and AP2 correspond to the mouse homologs. TSS1 and TSS2 represents the upstream and downstream translation start sites, respectively.

b) Phylogenetic distribution profiles of PRPS homologs (PRPS1, PRPS2, PRPSAP2, PRPSAP2 with N-terminal leader sequence, PRPSAP1, and PRPSAP1 with N-terminal leader sequence) in chordates (presence/absence) are noted across the tree. PRPSAP2 and PRPSAP1 isoforms with additional N-terminal leader sequences emerged in the ancestor of Amniota and Osteichthyes, respectively.

c) and d) The N-terminal residues from a sequence alignment of PRPSAP2 (c) and PRPSAP1 (d) from representative organisms of Amniota and Osteichthyes, respectively. TSS1 and TSS2 represents the upstream and downstream translation start sites, respectively.

bioRxiv preprint doi: https://doi.org/10.1101/2024.10.01.616059; this version posted October 1, 2024. The copyright holder for this preprint (which was not certified by peer review) is the author/funder, who has granted bioRxiv a license to display the preprint in perpetuity. It is made available under aCC-BY 4.0 International license.

Supplementary Fig. 10

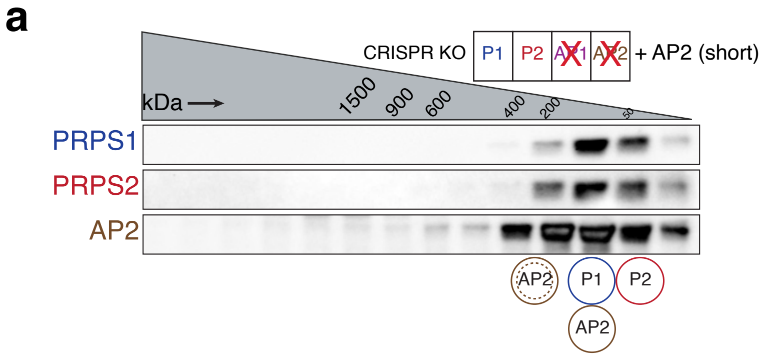

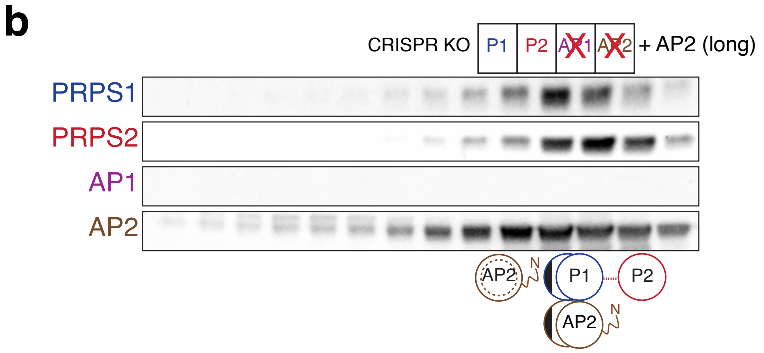

bioRxiv preprint doi: https://doi.org/10.1101/2024.10.01.616059; this version posted October 1, 2024. The copyright holder for this preprint (which was not certified by peer review) is the author/funder, who has granted bioRxiv a license to display the preprint in perpetuity. It is made available under aCC-BY 4.0 International license.

Supplementary Fig.10: SEC profiles of long and short PRPSAP2 isoforms

a) and b) Western blot analysis of SEC fractions collected from NIH3T3 AP1/AP2 KO cells stably expressing the short isoform of AP2 (a) and long isoform of AP2 (b). Cell lysates were fractionated on a Superose 6 Increase 3.2/300 column.

The circular pictograms at the bottom of the SEC immunoblots illustrate the different configurations of the PRPS complex. A double circle with a dotted inner circle means multiple copies of the protein are forming homo-oligomers. A single circle means a single protein is interacting within the complex. A circle with lines inside indicates that the proteins may be forming a trimer or tetramer.

bioRxiv preprint doi: https://doi.org/10.1101/2024.10.01.616059; this version posted October 1, 2024. The copyright holder for this preprint (which was not certified by peer review) is the author/funder, who has granted bioRxiv a license to display the preprint in perpetuity. It is made available under aCC-BY 4.0 International license.

Supplementary Fig.11

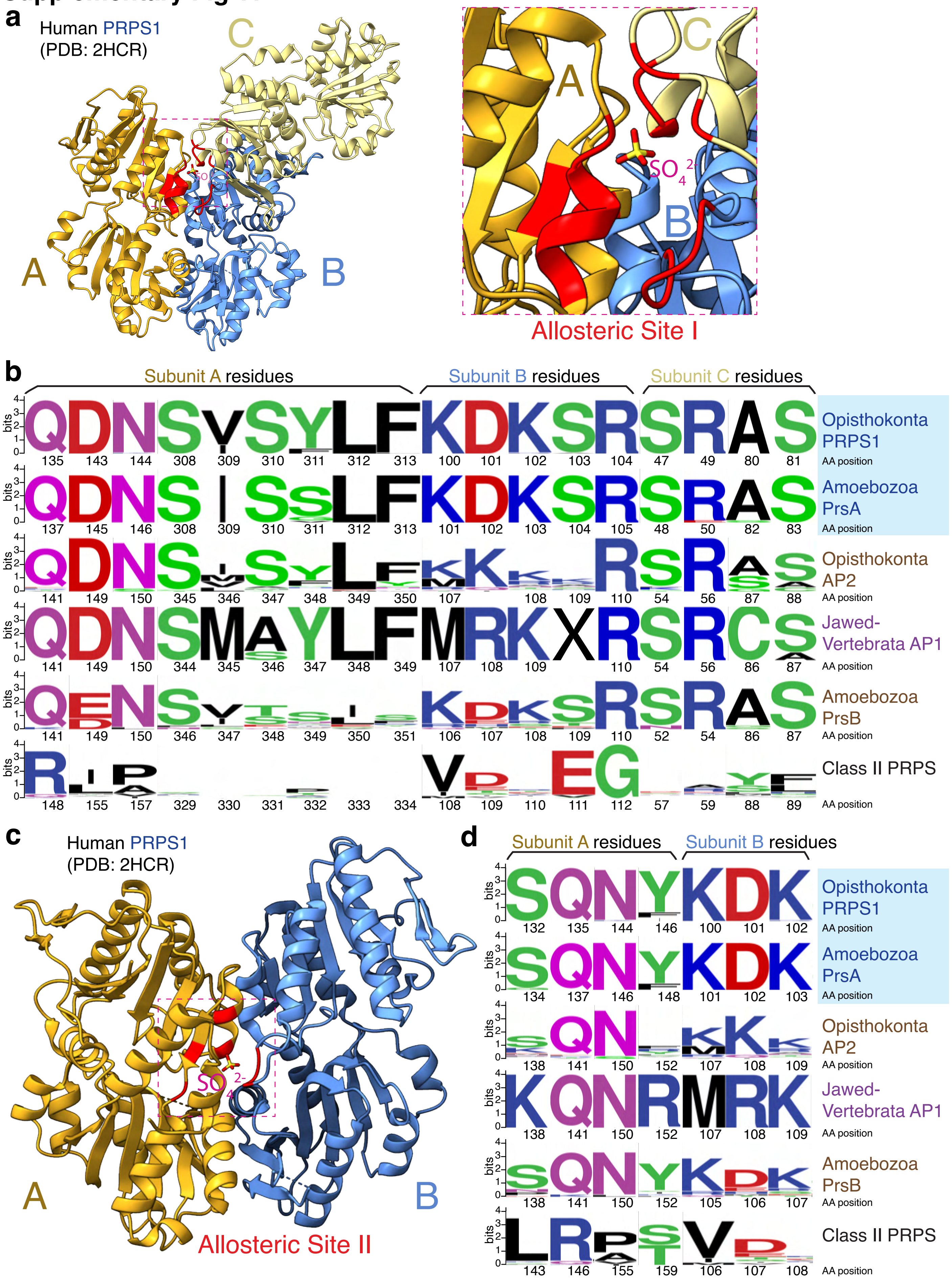

bioRxiv preprint doi: https://doi.org/10.1101/2024.10.01.616059; this version posted October 1, 2024. The copyright holder for this preprint (which was not certified by peer review) is the author/funder, who has granted bioRxiv a license to display the preprint in perpetuity. It is made available under aCC-BY 4.0 International license.

Supplementary Fig.11: Comparison of allosteric site across PRPS homologs

a) The structure of trimeric human PRPS1 (PDB ID: 2HCR). The dashed box represents the allosteric site I, a zoom in of this site shows SO_4^{2} (which represents the phosphate of ADP) positioned at the trimeric interface and red color indicates the residues from each subunit contributing to the formation of allosteric site I.

b) The amino acid sequence of the human PRPS1 was aligned with sequences of representative organisms from Opisthokonta PRPS1 (n = 44), Amoebozoa PrsA (n = 19), Opisthokonta PRPSAP2 (n = 46), jawed Vertebrata PRPSAP1 (n = 92), Amoebozoa PrsB (n = 20) and Class II PRPS (n = 53), and the corresponding allosteric site I residues were selected for generating the WebLogo. The numbers below the logo sequences indicate the corresponding residues positions of human PRPS1 and PRPSAP2 (for Opisthokonts), *R. potamoides* PrsA and PrsB (for Amoebozoa), human PRPSAP1 (for jawed Vertebrata), and *A. parasiticum* Class II PRPS annotated from SRX179384. X represents the absent amino acid residue at that position jawed Vertebrata AP1. Class II PRPS enzymes are shown as a reference since they are known to lack allosteric sites found in Class I PRPS ("classical" PRPS enzymes)[2].

c) The structure of dimeric human PRPS1 (PDB ID: 2HCR). The dashed box represents the allosteric site II, a zoom in of this site shows SO_4^{2} positioned at the dimer interface and red color indicates the residues from each subunit contributing to the formation of allosteric site II.

d) The amino acid sequence of the human PRPS1 was aligned with sequences from Opisthokonta PRPS1, Amoebozoa PrsA, Opisthokonta PRPSAP2, jawed Vertebrata PRPSAP1, Amoebozoa PrsB and Class II PRPS, and the corresponding allosteric site II residues were selected for generating the WebLogo similar to (b).

bioRxiv preprint doi: https://doi.org/10.1101/2024.10.01.616059; this version posted October 1, 2024. The copyright holder for this preprint (which was not certified by peer review) is the author/funder, who has granted bioRxiv a license to display the preprint in perpetuity. It is made available under aCC-BY 4.0 International license.

Supplementary Fig. 12

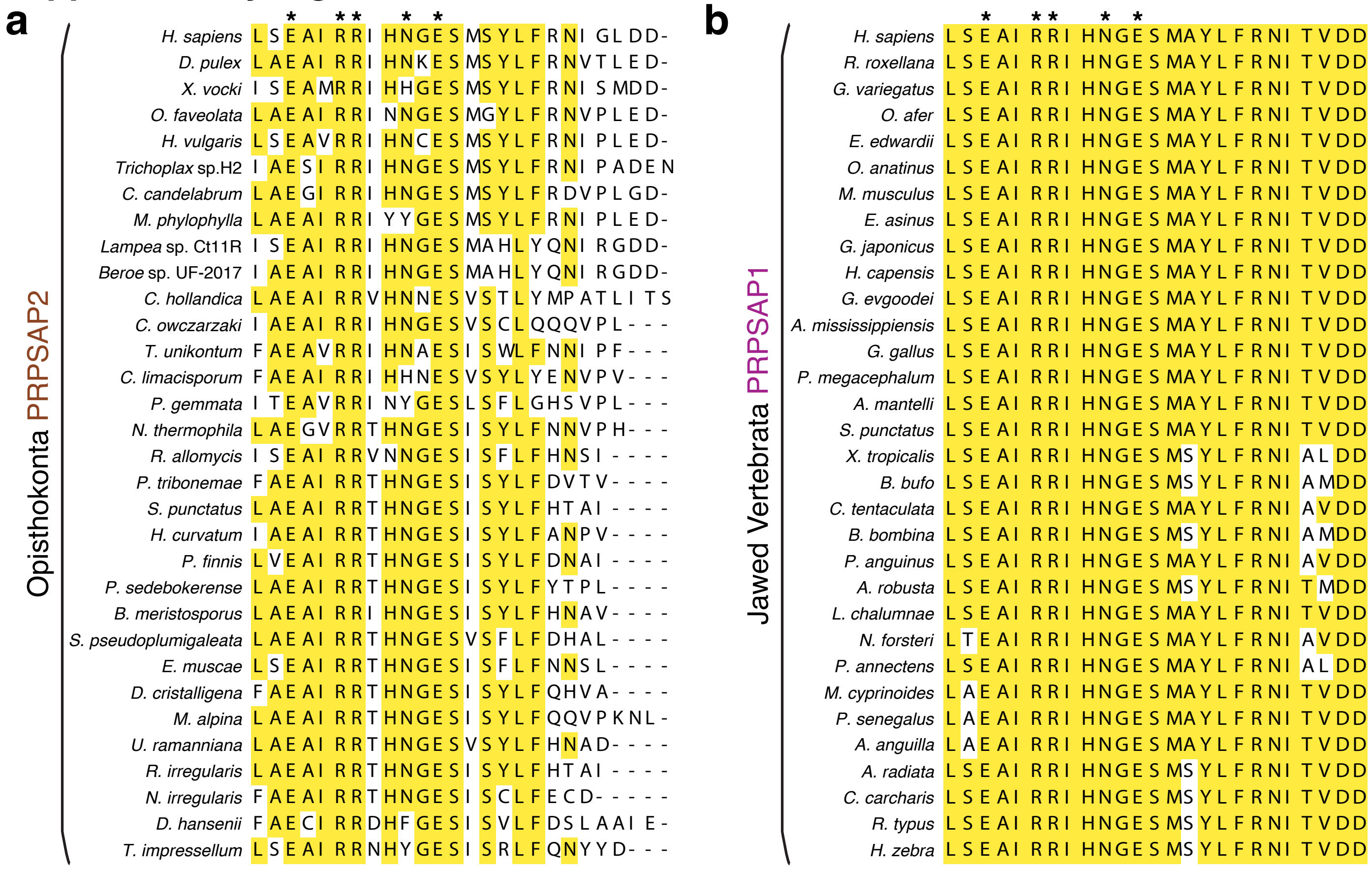

bioRxiv preprint doi: https://doi.org/10.1101/2024.10.01.616059; this version posted October 1, 2024. The copyright holder for this preprint (which was not certified by peer review) is the author/funder, who has granted bioRxiv a license to display the preprint in perpetuity. It is made available under aCC-BY 4.0 International license.

Supplementary Fig.12: Conservation of filament interfaces in PRPSAPs

a) and b) The C-terminal residues from the sequence alignments of representative organisms from Opisthokonta PRPSAP2 (a) and jawed Vertebrata PRPSAP1 (b). Residues, marked with an asterisk, critical for hexamer stacking at the filament interface are deeply conserved in PRPSAPs.

bioRxiv preprint doi: https://doi.org/10.1101/2024.10.01.616059; this version posted October 1, 2024. The copyright holder for this preprint (which was not certified by peer review) is the author/funder, who has granted bioRxiv a license to display the preprint in perpetuity. It is made available under aCC-BY 4.0 International license.

Supplementary Fig. 13

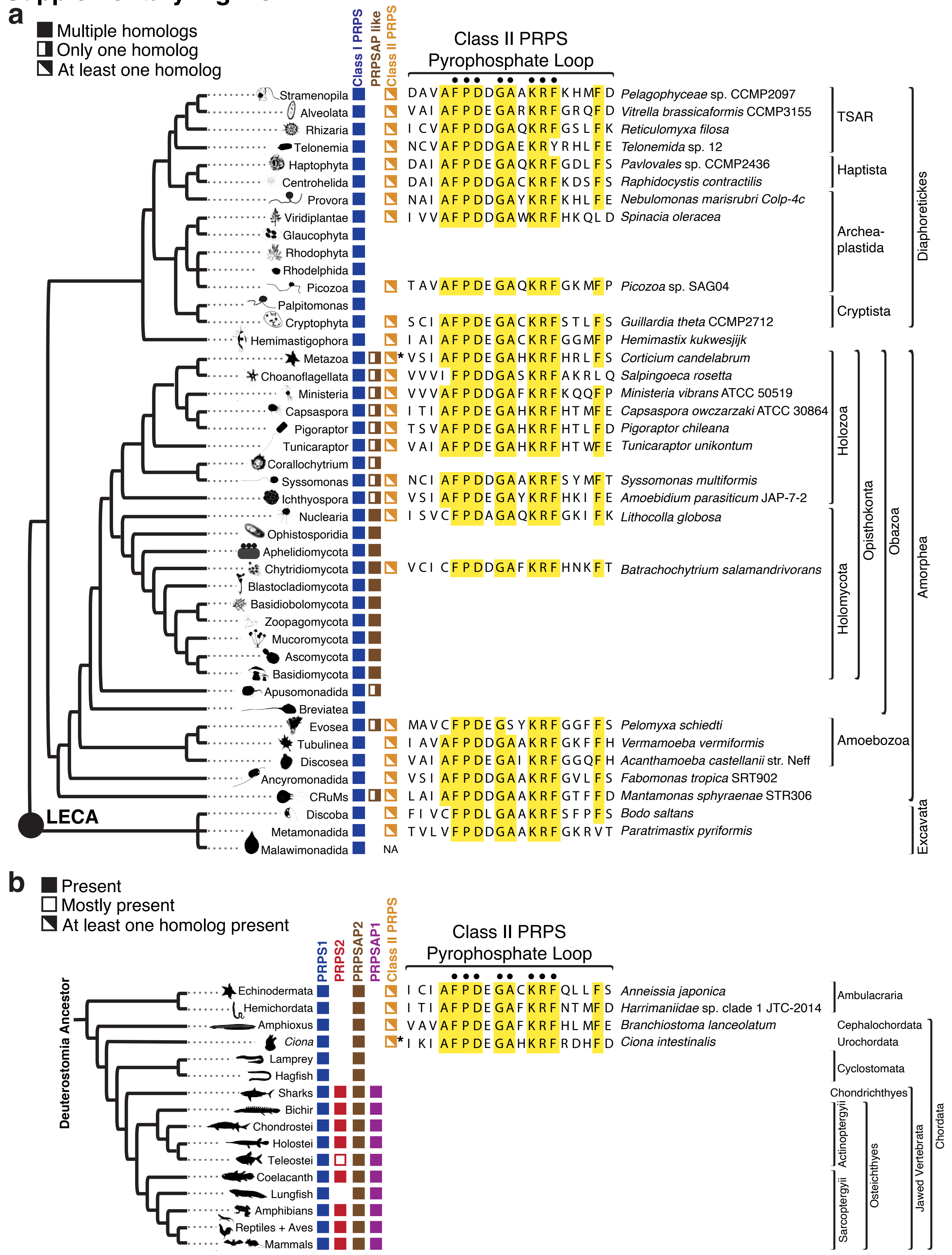

bioRxiv preprint doi: https://doi.org/10.1101/2024.10.01.616059; this version posted October 1, 2024. The copyright holder for this preprint (which was not certified by peer review) is the author/funder, who has granted bioRxiv a license to display the preprint in perpetuity. It is made available under aCC-BY 4.0 International license.

Supplementary Fig.13: Loss of Class II PRPS enzyme associates with expanded Class I PRPS homolog repertoire.

a) Eukaryotic phylogenetic tree of members of Class I PRPS and PRPAP-like as well as Class II PRPS. The presence of single or multiple homologs, that we have detected so far, are noted across the tree. PRPSAP-like orthologs are categorized based on non-conserved active site residues and insertions of varied length in the CF loop. Asterisk in Metazoan Class II PRPS enzyme indicates that it is present in most metazoans except Craniata. Majority of Holomycota species lost Class II PRPS, that do not possess allosteric sites, while expanding multiple Class I PRPS homologs. Sequence alignment of residues surrounding pyrophosphate (PP) loop of class II PRPS for representative organisms of each clade are shown. Conserved residues indicated by solid dots are unique to Class II PRPS.

b) Phylogenetic distribution profiles of Class I (PRPS1, PRPS2, PRPSAP1, PRPSAP2) and Class II in deuterostomes (presence/absence) are noted across the tree. In the Urochordata class, Class II PRPS is generally found in most organisms, except for *Oikopleura* which interestingly possess additional Class I PRPS homologs compared to other organisms in this clade. Sequence alignment of residues surrounding pyrophosphate (PP) loop of class II PRPS for representative organisms of each clade is shown. Conserved residues indicated by solid dots are unique to Class II PRPS enzymes. Following the loss of Class II PRPS in Cyclostomata, PRPS2 and PRPSAP1 emerged from gene duplications events in the ancestor of jawed vertebrates.

bioRxiv preprint doi: https://doi.org/10.1101/2024.10.01.616059; this version posted October 1, 2024. The copyright holder for this preprint (which was not certified by peer review) is the author/funder, who has granted bioRxiv a license to display the preprint in perpetuity. It is made available under aCC-BY 4.0 International license.

Supplementary Table.1: Comparion of active site residues of *B. subtilis* PRPS with corresponding amino acid residues of other Class I PRPS homolgs

Bacteria		Archaea	CRuMs		Apusozoa		Amoebozoa				Opisthokonta			
			D. rotans		*T. trahens*		*R. potamoides*		*D. discoideum*		*H. sapiens*			
B. subtilis	*E. coli*	*M. jannaschii*	PRPS	AP-like	PRPS	AP-like	PrsA	PrsB	PrsA	PrsB	PRPS1	PRPS2	PRPSAP1	PRPSAP2
**FLAG region**														
F40	F35	F32	F36	H36	F39	F41	F36	F40	F36	F37	F35	F35	E42	E42
D42	D37	D34	D38	D38	D41	D43	N38	N42	N38	N39	N37	N37	N44	N44
E44	E39	E36	E40	E40	E43	E45	E40	E44	E40	E41	E39	E39	E46	E46
V45	V40	I37	C41	L41	R44	I46	T41	T45	T41	T42	T40	T40	T47	T47
**Regulatory flexible loop and ATP phosphate chain binding**														
Y99	Y94	Y90	Y95	Y95	Y99	Y101	Y96	Y100	Y96	Y97	Y94	Y94	Y101	Y101
T113	T119	S104	S109	S109	S113	A115	T110	T114	T110	F111	S108	S108	V114	V114
R101	R96	R92	R97	R97	R101	R102	R98	R102	R98	R99	R96	R96	K103	K103
Q102	Q97	Q93	Q98	Q98	A102	Q103	Q99	Q103	Q99	Q100	Q97	Q97	Q104	Q104
D103	D98	D94	D99	D99	D103	D104	D100	S104	D100	S101	D98	D98	S105	C105
R104	D99	K95	R100	R100	R104	T105	K101	K105	K101	K102	K99	K99	K106	K106
H135	H131	H125	H131	H131	H135	H137	H132	H136	H132	H133	H130	H130	H136	H136
**Pentose phosphate loop (PP loop)**														
D174	D170	D163	D171	D176	D177	S182	D171	G179	D171	G177	D171	D171	S177	S177
G176	G172	G165	G173	G178	G179	K184	G173	G181	G173	G179	G173	G173	D179	A179
G177	G173	A166	G174	S179	G180	A185	G174	G182	G174	G180	G174	G174	A180	S180
**Catalytic flexible loop**														
K197	K194	K186	K200	K199	K205	R210	K194	R202	K196	R200	K194	K194	G200	G200
R199	R196	K188	R202	S200	R207	V212	R196	N204	R196	N202	R196	R196	A202	A202
N203	N200	T192	G206	T205	G211	Q216	N200	L208	N200	S206	N200	N200	E206	E206
**Ribose 5-phosphate binding loop**														
D223	D220	D212	D228	D306	D233	D254	D220	D258	D200	D235	D220	D220	D256	D257
D224	D221	D213	D229	D307	D234	D255	D221	D259	D221	D236	D221	D221	D257	D258
D227	D224	S216	D232	D310	D237	F258	D224	D262	D224	D239	D224	D224	D260	D261
T228	T225	T217	T233	T311	T238	S259	T225	T263	T225	T240	T225	T225	D261	D262
A229	G226	G218	A234	G312	C239	G260	C226	C264	C226	C241	C226	C226	V262	V263
T231	T228	T220	T236	T314	T241	T262	T228	T266	T228	T243	T228	T228	S264	S265
I232	L229	M221	L237	I315	L242	L263	L229	L267	L229	L244	I229	I229	F265	F266

The annotated amino acid residues of the active site of B. subtilis PRPS[15] enzyme are listed based on their functions. Amino acid residues at similar positions based on pairwise alignment in PRPS of E. coli (Accession #U00096) ; PRPS of M. jannaschii (Accession #L77117); PRPS (annotated from SRX3153023) and PRPSAP-like (annotated from SRX3153023) of D. rotans; PRPS (XP_013753676.1) and PRPSAP-like (XP_013760460.1) of T. trahens; PrsA (annotated from SRX8374346-9) and PrsB (annotated from SRX8374346-9) of R. potamoides; PrsA (XP_638466.1) and PrsB (XP_645319.1) of D. discoideum; PRPS1 (NP_002755.1), PRPS2 (NP_002756.1), PRPSAP1 (AAH09012.1), and PRPSAP2 (NP_001340030.1) of human are shown. Non-conserved residues relative to B. subtilis PRPS enzyme are highlighted in magenta. Active site residues appear to be poorly conserved in PRPSAP-like, PrsB, PRPSAP1 and PRPSAP2 proteins.
